# Do comorbidities influence help-seeking for cancer alarm symptoms? A population-based survey in England

**DOI:** 10.1093/pubmed/fdx072

**Published:** 2017-06-24

**Authors:** Theodosia Salika, Georgios Lyratzopoulos, Katriina L Whitaker, Jo Waller, Cristina Renzi

**Affiliations:** 1 Department of Behavioural Science and Health, University College London, 1-19 Torrington Place, London, UK; 2 School of Health Sciences, University of Surrey, Surrey, UK

**Keywords:** cancer, comorbidity, diagnosis, help-seeking, symptoms

## Abstract

**Background:**

We examined associations between different chronic morbidities and help-seeking for possible cancer symptoms.

**Methods:**

Postal survey of individuals aged >50 years in England. Participants could report prior morbidities in respect of 12 pre-defined conditions. Among patients experiencing possible cancer symptoms we examined associations between specific morbidities and self-reported help-seeking (i.e. contacted versus not contacted a GP) for each alarm symptom using regression analyses.

**Results:**

Among 2042 respondents (42% response rate), 936 (46%) recently experienced 1 of 14 possible cancer symptoms considered in our analysis. Of them, 80% reported one or more morbidities, most frequently hypertension/hypercholesterolemia (40%), osteomuscular (36%) and heart diseases (21%). After adjustment for socio-demographic characteristics, patients with hypertension/hypercholesterolemia were more likely to report help-seeking for possible cancer symptoms, such as unexplained cough (OR = 2.0; 95% confidence interval (CI) 1.1–3.5), pain (OR = 2.2; 95% CI 1.0–4.5) and abdominal bloating (OR = 2.3; 95% CI 1.1–4.8). Urinary morbidity was associated with increased help-seeking for abdominal bloating (OR = 5.4; 95% CI 1.2–23.7) or rectal bleeding (OR = 5.8; 95% CI 1.4–23.8). In contrast, heart problems reduced help-seeking for change in bowel habits (OR = 0.4; 95% CI 0.2–1.0).

**Conclusions:**

Comorbidities are common and may facilitate help-seeking for possible cancer symptoms, but associations vary for specific symptom-comorbidity pairs. The findings can contribute to the design of future cancer symptom awareness campaigns.

## Introduction

Chronic conditions affect large proportions of older individuals. Data from both Scotland and England indicate that about half of the general population have a long-standing health condition, of whom half have two or more morbidities.^1,2^ As older age is associated with both a higher prevalence of chronic diseases and higher cancer risk, it is important to examine the likely influence of pre-existing morbidities (hereafter called comorbidities) on help-seeking behavior for new symptoms that may relate to cancer. Given the high morbidity prevalence, even small effects can have important consequences at population level.

Although some studies indicate that comorbidities may lead to more prompt help-seeking in patients with upper^3–5^ and lower gastrointestinal cancers^6^ and no delay for lung cancer symptoms^7,8^; other studies suggest that comorbidities can delay as well as facilitate help-seeking, with various factors possibly coming into play, including the type of comorbidity and symptom characteristics (e.g. evolution of symptoms over time or their severity).^9,10^ Mechanisms underlying the possible association between comorbidity and help-seeking have only been partially explored. Comorbidities may enable the reporting of possible cancer symptoms during healthcare encounters for the chronic problem. Sometimes patients might feel that help-seeking for vague symptoms is only appropriate if the consultation is also needed for a co-existing morbidity.^11^ Familiarity with the healthcare provider and/or system may also facilitate prompt help-seeking. Such mechanisms are supported by patients’ accounts in qualitative interviews^12^ and are in line with higher consultation rates among patients with chronic health problems .^13^

On the other hand, comorbidities may lead to delays by interfering with symptom appraisal and/or help-seeking if the management of the chronic disease(s) is perceived as more important than the new symptom, particularly if the new symptoms are vague and do not interfere with daily life.^10^ Similarly, patients (and their carers) may attribute new symptoms to pre-existing disease(s), delaying help-seeking or delaying reporting of potential cancer symptoms.^12,14–16^

Complex interactions between specific types of morbidity and the nature of the potential cancer symptoms are likely. For example, some lung cancer patients reported pre-existing respiratory and cardiac comorbidities as reasons for delayed help-seeking,^9,14^ but others were able to identify the ‘unusual or changing symptoms’ despite chronic respiratory problems.^9^

Despite these complex mechanisms and possible interactions between different types of comorbidities and symptom characteristics, the majority of population-based studies have only reported on the overall effect of comorbidity, with only a few studies examining specific comorbidities.

Against this background, our objective was to evaluate the associations between specific comorbidities and help-seeking for different cancer alarm symptoms among a population sample. Our broader aim was to produce evidence that can contribute to the design of future public health educational interventions (e.g. ‘awareness campaigns’) for improving early diagnosis of cancer.

## Methods

A health survey was mailed in October 2013 to 4913 individuals aged ≥50 years in England from four general practices and 2042 questionnaires were returned (response rate 42%). Details of the study methods have been previously described.^17,18^ Exclusion criteria were a previous cancer diagnosis and severe physical or mental health problems based on primary care records. Participants were asked to report if they experienced any of 14 ‘alarm’ symptoms for possible cancer in the last 3 months. The symptom list was based on the Cancer Awareness Measure^19^ and Be Clear on Cancer Campaigns.^20^

Participants were asked whether they had contacted their GP for each of the symptoms experienced during the last 3 months and the time between symptom onset and help-seeking. For the present study, we dichotomized help-seeking, with our primary help-seeking outcome being defined as having contacted the GP for the experienced symptom (independently of the time interval between symptom onset and help-seeking) versus not having contacted the GP (see Box 1). As information on help-seeking was reported separately for each recently experienced symptom, all analyses were stratified by symptom category (i.e. 14 different strata). In addition, we dichotomized the speed of help-seeking into ‘sought help within 3 months from symptom onset’ versus ‘did not seek help for 3 or more months from symptom onset’. Prompt help-seeking (within 3 months from symptom onset) versus non-prompt help-seeking was examined as a secondary outcome.

Box 1 **Survey questions**

1. **In the last 3 months, have you had the following…?** (indicate yes/no for each of the following) Response categories (14): Unexplained weight loss, unexplained lump, change in the appearance of a mole or a new mole, persistent change in bowel habits, blood in urine, persistent change in bladder habits, any breast changes, persistent unexplained pain, persistent difficulty swallowing, persistent cough or hoarseness, rectal bleeding (i.e. bleeding from the back passage or blood in the bowel motions), other unexplained bleeding, abdominal bloating (i.e. bloating of your tummy or belly), a sore that does not heal.
2. **Approximately how long after the symptom began did you contact the GP about it?** Response categories: a) Did not contact the GP, b) Not contacted the GP yet but plan to, c) within 1–2 weeks of noticing the symptom, d) within 1 month of noticing the symptom, e) within 6 weeks of noticing the symptom, f) within 3 months of noticing the symptom, g) after >3 months of noticing the symptom. [Our primary outcome included response categories a–b denoting no help-seeking versus response categories c–g denoting help-seeking. For our secondary outcome, i.e. prompt help-seeking (within 3 months from symptom onset), response categories c–f were treated as denoting prompt help-seeking versus response categories a, b and g as denoting non-prompt help-seeking].
3. **Do you have a diagnosis of any of the following conditions/illnesses?** Response categories (1): arthritis, cancer, circulation problems, chest problems, cholesterol problems, depression, diabetes, heart problems, high blood pressure, kidney problems, stroke, other (please specify-in free-text). The above pre-coded and free-text responses were further aggreagated into 10 new morbidity categories in order to have sufficient numbers per category for further analyses, comprising: hypertension/hypercholesterolemia, osteomuscular, respiratory, heart problems, mental health (depression and other free-text mental health problems), diabetes/endocrinological, neurological, urinary, gastrointestinal problems and others (free-text responses such as skin, eye and hearing problems).

Information on comorbidities was based on respondents’ replies to the question ‘Do you have a diagnosis of any of the following conditions/illnesses?’ (Tick all that apply/if other, please specify). This encompassed 12 pre-coded morbidity categories including a free-text response option for adding any other morbidity (see Box 1). Using the structured and free-text responses we defined ten morbidity categories that were included in further analyses: hypertension/hypercholesterolemia, osteomuscular, respiratory, heart, mental health, diabetes/endocrinological, neurological, urinary, gastrointestinal problems and others.

### Analysis

We examined the most frequent single, dual and triple symptom and comorbidity combinations. Because of sample size limitations, further analyses were restricted to the seven most frequent symptoms (*n* > 100), i.e. persistent cough or hoarseness, persistent unexplained pain, abdominal bloating, change in the appearance of mole, persistent change in bowel habits, persistent change in bladder habits and rectal bleeding.

Within each symptom stratum, we examined univariable associations between each specific comorbidity and help-seeking for the specific symptom and socio-demographic factor (age, sex and educational level). Smoking status was also included in univariable analysis among respondents with a cough. Subsequently, we performed multivariable logistic regression examining the association between specific comorbidities and help-seeking, controlling for socio-demographic characteristics. A separate model was used for each cancer alarm symptom. Each final model included variables thought a priori to be potentially associated with help-seeking (sex, age and educational level) and those comorbidities associated with help-seeking in univariable analysis at *P*-value <0.2. All analyses were performed for our main outcome of interest (help-seeking versus non help-seeking) and subsequently repeated for our secondary outcome (prompt versus non-prompt help-seeking).

In order to limit the possibility of reverse causation bias, we restricted our analyses a priori to comorbidity-symptom pairs with non-overlapping clinical presentations, such as chronic conditions affecting body systems or organs unrelated to the specific symptom/sign (e.g. pre-existing chronic obstructive pulmonary disease (COPD) and change in the appearance of a skin mole) or asymptomatic comorbidities (e.g. hypertension or hypercholesterolemia). Consequently, we excluded from our analyses morbidity-symptom pairs such as COPD and cough or osteomuscular problems and pain.

STATA 14 was used for statistical analyses and the significance level was set at *P* < 0.05.

## Results

Out of 2042 respondents 936 (46%) had experienced at least one cancer alarm symptom during the last 3 months. After excluding 21 individuals who reported a previous cancer diagnosis in the free-text responses, 915 symptomatic participants were available for analysis. Both symptomatic and asymptomatic participants had a median age of 64 years (interquartile range (IQR):57–71, and IQR: 57–70, respectively). Symptomatic participants reported a median of one symptom (IQR: 1–2) and two comorbidities (IQR: 1–3) whereas asymptomatic participants reported one comorbidity (IQR: 1–2).

Subsequent analyses refer only to symptomatic participants. The most frequently reported symptoms included persistent cough or hoarseness (30%; *n* = 272), persistent unexplained pain (23%; *n* = 213), abdominal bloating (23%; *n* = 210), change in bowel habits (18%; *n* = 167) and persistent change in bladder habits (17%; *n* = 158). The most frequent comorbidities were hypertension/hypercholesterolemia (40%; *n* = 368), osteomuscular (36%; *n* = 330) and heart diseases (21%; *n* = 189) (Table 1).

**Table 1:** Demographic characteristics among symptomatic and asymptomatic participants

		Symptomatic (*N* = 915) *n* (%)	Asymptomatic (*N* = 1106) *n* (%)
Sex	Males	373 (40.8)	551 (49.8)
	Females	534 (58.4)	543 (49.1)
Education	Higher education	341 (37.3)	389 (35.2)
	Higher education below degree/A level	164 (17.9)	212 (19.2)
	ONC/BTCE/O Level/GCSE	143 (15.6)	172 (15.6)
	No formal qualifications	170 (18.6)	251 (22.7)
	Other	75 (8.2)	62 (5.6)
Smoking status	No, I have never smoked	408 (44.6)	522 (47.2)
	Not now, but I used to smoke	378 (41.3)	451 (40.8)
	Yes, I smoke occasionally	28 (3.1)	43 (3.9)
	Yes, I am a current smoker	96 (10.5)	82 (7.4)
Cancer symptom	Persistent cough or hoarseness	272 (29.7)	
	Persistent unexplained pain	213 (23.3)	
	Abdominal bloating	210 (23.0)	
	Change in bowel habits	167 (18.3)	
	Change in bladder habits	158 (17.3)	
	Rectal bleeding	145 (15.8)	
	Change in a mole	144 (15.7)	
	Unexplained lump	72 (7.9)	
	Unexplained weight loss	68 (7.4)	
	A sore that does not heal	55 (6.0)	
	Blood in urine	48 (5.2)	
	Persistent difficulty swallowing	47 (5.1)	
	Any breast changes	31 (3.4)	
	Other bleeding	23 (2.5)	
Comorbidity	Hypertension/hypercholesterolemia	368 (40.2)	401 (36.3)
	Osteomuscular problems	330 (36.1)	232 (21.0)
	Heart problems	189 (20.7)	145 (13.1)
	Respiratory problems	126 (13.8)	51 (4.6)
	Mental health problems	110 (12.0)	58 (5.2)
	Diabetes/thyroid	87 (9.5)	86 (7.8)
	Neurological problems	69 (7.5)	23 (2.1)
	Urinary problems	62 (6.8)	24 (2.2)
	Gastrointestinal problems	55 (6.0)	0 (0.0)
	Other (a)	63 (6.9)	223 (20.2)
	No comorbidity	187 (20.4)	359 (32.5)

Numbers may not add up to the total due missing data for some variables. For symptoms and comorbidity totals exceed 100% due to multiple symptoms and comorbidities.

(a) Other comorbidity; skin, eye, hearing problems etc.

Approximately half of symptomatic participants (56%) reported one symptom, 24% two and 11% three symptoms. A single comorbidity was reported by 33% of symptomatic participants, while 25 and 12% reported dual or triple comorbidities, respectively. The prevalence of each specific comorbidity reported either as a single comorbidity or in combination with other comorbidities is shown in Table 2. The most frequent dual comorbidity combination was osteomuscular problems and hypertension/hypercholesterolemia (further details in Appendix). The combination of osteomuscular, hypertension/hypercholesterolemia and heart problems was the most frequent triple morbidity (2%; *n* = 17).

**Table 2:** Prevalence of each comorbidity as a single comorbidity or in combination with other comorbidities (dual and triple morbidities) among symptomatic individuals (Total *N* = 915)

	Overall prevalence (as single or multiple morbidity) (a)	Single morbidity	Dual morbidity	Triple morbidity	Four or more morbidities
	% (*n*)	% (*n*)	% (*n*)	% (*n*)	% (*n*)
Hypertension/hypercholesterolemia	40.2 (368)	8.7 (80)	14.9 (136)	9.0 (82)	7.6 (70)
Osteomuscular	36.1 (330)	8.5 (78)	12.6 (115)	7.7 (70)	7.3 (67)
Heart	20.7 (189)	2.8 (26)	5.8 (53)	3.3 (30)	8.7 (80)
Respiratory	13.8 (126)	2.6 (24)	3.8 (35)	2.7 (25)	4.6 (42)
Mental Health	12 (110)	1.7 (16)	4.3 (39)	2.2 (20)	3.8 (35)
Diabetes/Thyroid	9.5 (87)	1.2 (11)	2.2 (20)	3.4 (31)	2.7 (25)
Neurological	7.5 (69)	1.3 (12)	1.5 (14)	1.9 (17)	2.8 (26)
Urinary	6.8 (62)	2.1 (19)	1.1 (10)	1.5 (14)	2.1 (19)
Gastrointestinal	6 (55)	2.0 (18)	1.7 (16)	1.0 (9)	1.3 (12)
Other	6.9 (63)	2.3 (21)	2.4 (22)	1.0 (9)	1.2 (11)

(a) The numbers reported in the first column re-iterate some of the data reported in Table 1 and are shown here for completeness.

### Help-seeking for possible cancer symptoms—univariable analysis

Subsequent analyses were stratified according to the most frequent symptom categories. The proportion of participants having contacted their GP for their symptoms was 62% for unexplained pain, 54% for change in bowel habits, 48% for change in bladder habits, 46% for chronic cough, 39% for changes in a mole and 36% for rectal bleeding. Similarly prompt help-seeking (within 3 months from symptom onset) ranged from 48% for unexplained pain to 25% for rectal bleeding. Help-seeking for persistent cough or unexplained pain was associated with hypertension/hypercholesterolemia in univariable analysis (Table 3). Among participants experiencing abdominal bloating help-seeking was associated with having osteomuscular problems and hypertension/hypercholesterolemia. For patients with rectal bleeding help-seeking was associated with urinary comorbidities.

**Table 3:** Univariable logistic regression for the association between each specific comorbidity and help-seeking for possible cancer symptoms

Crude OR of help-seeking (95% CI) *P*-value							
	Persistent cough or hoarseness (*n* = 235)	Persistent unexplained pain (*n* = 184)	Abdominal Bloating (*n* = 172)	Change in bowel habits (*n* = 142)	Change in bladder habits (*n* = 134)	Change in a mole or a new mole (*n* = 127)	Rectal bleeding (*n* = 117)
Comorbidity							
Osteomuscular	1.7 (1.0, 2.9) *P* = 0.05	(a)	**2.1 (1.1, 4.0) *P* = 0.03**	1.4 (0.7, 2.9) *P* = 0.30	1.5 (0.8, 3.1) *P* = 0.23	0.6 (0.3, 1.3) *P* = 0.15	1.8 (0.8, 3.8) *P* = 0.13
Hypertension/hypercholesterolemia	**2.3 (1.4, 3.9) *P* = 0.002**	**2.2 (1.1, 4.2) *P* = 0.02**	**2.8 (1.4, 5.4) *P* < 0.01**	1.0 (0.5, 2.0) *P* = 0.95	1.4 (0.7, 2.8) *P* = 0.36	1.8 (0.9, 3.7) *P* = 0.12	1.6 (0.8, 3.5) *P* = 0.21
Mental health	1.2 (0.5, 2.8) *P* = 0.7	(a)	(a)	1.1 (0.5, 2.6) *P* = 0.84	1.2 (0.5, 3.0) *P* = 0.73	0.9 (0.3, 2.8) *P* = 0.83	1.3 (0.4, 4.4) *P* = 0.64
Respiratory	(a)	0.6 (0.3,1.6) *P* = 0.32	1.57 (0.6, 3.8) *P* = 0.32	0.6 (0.2, 1.8) *P* = 0.38	0.5 (0.2, 1.6) *P* = 0.25	0.3 (0.1, 1.0) *P* = 0.06	1.3 (0.4, 4.4) *P* = 0.64
Heart problems	0.9 (0.5, 1.7) *P* = 0.78	1.3 (0.6, 3.0) *P* = 0.56	1.58 (0.8, 3.3) *P* = 0.21	0.6 (0.3, 1.3) *P* = 0.18	1.1 (0.5, 2.4) *P* = 0.80	0.7 (0.3, 2.0) *P* = 0.54	1.5 (0.7, 3.7) *P* = 0.33
Diabetes/ Thyroid	1.6 (0.7, 4.0) *P* = 0.27	1.0 (0.4, 2.8) *P* = 1.00	(a)	2.4 (0.8, 7.9) *P* = 0.13	3.2 (0.7, 16.2) *P* = 0.15	0.5 (0.1, 3.1) *P* = 0.45	2.1 (0.6, 7.8) *P* = 0.28
Urinary	1.4 (0.6, 3.4) *P* = 0.48	4.4 (0.5, 35.5) *P* = 0.17	**5.8 (1.5, 22.6) *P* = 0.01**	1.4 (0.5, 4.4) *P* = 0.52	(a)	0.4 (0.1, 2.1) *P* = 0.25	**4.5 (1.2, 17.6) *P* = 0.03**
Neurological	1.3 (0.5, 3.2) *P* = 0.63	(a)	(a)	(a)	(a)	1.4 (0.4, 4.9) *P* = 0.63	1.0 (0.2, 4.5) *P* = 0.97
Gastrointestinal	0.4 (0.1, 1.7) *P* = 0.21	0.5 (0.2, 1.4) *P* = 0.19	2.1 (0.9, 5.2) *P* = 0.10	(a)	0.6 (0.1, 2.9) *P* = 0.56	1.6 (0.3, 8.8) *P* = 0.62	(a)

Bold represents associations at *P* < 0.05.

(a) Excluded pairs where symptoms might be due to the morbidity (e.g. ‘COPD-cough; osteomuscular-pain’).

### Multivariable analyses for the association between comorbidities and help-seeking for cancer symptoms

Multivariable logistic regression confirmed most of the previously described associations between specific comorbidities and help-seeking for common cancer ‘alarm’ symptoms, adjusting for socio-demographic factors and the presence of multiple morbidities. In particular, hypertension/hypercholesterolemia was associated with a higher likelihood of help-seeking for patients experiencing cough (OR = 2.0; 95% confidence interval (CI) 1.1–3.5), persistent pain (OR = 2.2; 95% CI 1.1–4.5) or abdominal bloating (OR = 2.3; 95% CI 1.1–4.8). Urinary problems were associated with increased help-seeking for patients experiencing abdominal bloating (OR = 5.4; 95% CI 1.2–23.7) and rectal bleeding (OR = 5.8; 95% CI 1.4–23.8). Heart disease was the only comorbidity associated with a lower likelihood of help-seeking, specifically for patients experiencing change in bowel habits (OR = 0.4; 95% CI 0.2–1.0).

Secondary analyses examining the association between comorbidity and prompt help-seeking (within 3 months from symptom onset) versus non-prompt help-seeking confirmed the findings from our main analysis (secondary analysis in Appendix).

## Discussion

### Main findings of this study

In our population-based sample, the large majority of individuals who experienced a possible alarm symptom for cancer had at least one co-existing morbidity, most commonly hypercholesterolemia/hypertension, osteomuscular or heart problems. The presence of comorbidity, such as hypertension/hypercholesterolemia was significantly associated with increased help-seeking for commonly reported alarm symptoms, including persistent unexplained cough, unexplained pain and abdominal bloating, independently of socio-demographic characteristics. Urinary comorbidities increased help-seeking among patients with rectal bleeding and abdominal bloating. In contrast, having a heart comorbidiy decreased help-seeking for change in bowel habits.

### What is already known

Chronic conditions affect significant proportions of older individuals.^1,2^ Theoretical models acknowledge their potential effect on help-seeking for cancer symptoms and diagnostic delays.^21^ However, large studies to date have mainly evaluated the overall presence/absence and possibly the number of morbidities,^5,6,22,23^ with few surveys having evaluated specific types of comorbidities.^10,24,25^ Moreover, definitions and data collection methods vary widely between studies, ranging from summary scores (e.g. Charlson comorbidity index) based on medical records, to patient self-reports using open or pre-coded questions. Some qualitative patient interviews offer detailed information on specific comorbidities and their effect on help-seeking,^9^ but they are by nature limited to a small number of purposively selected patients.

Our findings are in line with prior studies examining factors influencing the time interval before cancer diagnosis among patients with upper^3–5^ and lower gastrointestinal cancers^6^ and with some patient interview studies showing that comorbidities can facilitate help-seeking or encourage reporting of possible cancer symptoms during medical encounters.^9^ Similarly, a study on emergency colorectal cancer diagnosis showed a higher prevalence of hypertension in non-emergency patients, suggesting that regular GP visits for blood pressure monitoring might have prevented a delayed cancer diagnosis.^25^ However, the overall evidence is mixed, with some other studies reporting no association between specific comorbidities (diabetes, heart and respiratory problems) and emergency colon cancer diagnosis^26^ or advanced stage at diagnosis.^23^

On the other hand, severe morbidities, such dementia, cerebrovascular disease, congestive heart failure and liver disease have been reported to be strongly associated with emergency colorectal cancer diagnosis.^27^ Cardiac and respiratory diseases have also been reported as reasons for delayed help-seeking among lung cancer patients.^9,14^ In some cases, comorbidities may lead to delays by interfering with symptom appraisal if the cancer symptom is attributed to the pre-existing condition.^12,14–16^

Therefore, prior evidence indicates that comorbidities may have complex effects, possibly acting both as barriers and as facilitators, depending on the specific type of morbidity and cancer symptom characteristics.

### What this study adds

Our study substantially amplifies the existing literature providing detailed information on the associations between specific comorbidity types and different cancer symptoms. A particular strength of our study is the inclusion of information on help-seeking for a range of common ‘alarm’ symptoms in the context of ten different comorbid conditions in a relatively large population-based sample. Having been able to examine specific comorbidity-cancer symptom pairs has allowed to appropriately characterize heterogeneity in the associations, with some comorbidities acting as barriers and others as facilitators.

Concordant with prior evidence,^3–6,9^ we found that osteomuscular morbidities and hypertension/hypercholesterolemia, which often require regular GP visits (for pain management or monitoring of blood pressure) facilitate help-seeking possibly by offering opportunities for discussing potential cancer symptoms in the context of ‘routine’ clinical encounters related to chronic disease management and monitoring. On the other hand, morbidities that are perceived as particularly serious and requiring more urgent attention, such as cardiac problems, seem to delay help-seeking in our study, as they might have led patients to postpone the discussion of other symptoms particularly if symptoms are vague or not interfering with daily life.

Our findings suggest that public health educational interventions for improving awareness of cancer symptoms and diagnose cancer earlier should take into account that individuals experiencing cancer symptoms often also have comorbidities, which affect symptom appraisal and help-seeking. Patients with comorbidities requiring regular GP visits (e.g. hypertension) might particularly benefit from holistic clinical management^28^ with clear guidance and encouraging them to report new symptoms when those occur.

Given the high prevalence of co-existing morbidity among patients with potential cancer symptoms further studies are needed to exactly understand how to improve early cancer diagnosis among comorbid patients.

## Limitations

Due to the cross-sectional study design and due to the fact that information on symptoms and comorbidity was self-reported we cannot exclude reverse causation between help-seeking and comorbidity, i.e. participants who sought help for symptoms might have been more likely to have received (or reported) a comorbidity diagnosis. However, we have limited this possible bias by only including in the analysis only comorbidity-symptom pairs that are unrelated physiologically or that affect different organs/systems and asymptomatic comorbidities. Longitudinal studies would be necessary to overcome this possible limitation. Severity of symptoms and comorbidity might also influence help-seeking, but this information was not available in our study.

It should also be noted that comorbidities may affect not only patients’ initial decisions to seek help for cancer symptoms, but they might also complicate the subsequent diagnostic process, influencing both patients and the healthcare providers during the different diagnostic phases. Significant event audits^28^ and patient interviews^9^ have shown that chronic morbidities might lead to missed opportunities to investigate symptoms suggestive of cancer or to patients delaying returning to the doctor after their symptoms were initially attributed to a co-existing morbidity.^12,14^ These factors were not examined in the present study and further research is warranted.

## Conclusions

Understanding the effect of co-existing morbidities on help-seeking in case of potential cancer symptoms is important, given that the vast majority of older individuals who experience an alarm symptom have chronic health problems. We reported how individuals with different morbidities may generally be more likely to seek help for common alarm symptoms compared to individuals without morbidity. These findings may be one of the many manifestations of ‘paradoxical benefits’ of morbidity. However, we have also shown that the effect might vary for specific symptom-comorbidity pairs and specific attention needs to be dedicated to some comorbidities, which might delay help-seeking. Interventions for improving early cancer diagnosis should take into account that most patients at risk of developing cancer will have at least one pre-existing morbidity with heterogeneous effects of common morbidities on help-seeking.

Larger quantitative studies are needed focusing on the effect of specific morbidities on help-seeking for different alarm symptoms and examining the influence of morbidity on doctors’ decision-making.

## Funding

This work was part of the programme of the Policy Research Unit in Cancer Awareness, Screening and Early Diagnosis. The Policy Research Unit in Cancer Awareness, Screening, and Early Diagnosis receives funding for a research programme from the Department of Health Policy Research Programme. It is a collaboration between researchers from seven institutions (Queen Mary University of London, University College London, King's College London, London School of Hygiene and Tropical Medicine, Hull York Medical School, Durham University and Peninsula Medical School). GL and TS were supported by Cancer Research UK Advanced Clinician Scientist Fellowship (C18081/A18180).

## Conflict of interest

None declared.

## Appendix

**Appendix 1:** Most frequent comorbidity combinations among individuals with dual comorbidity (percentages are calculated on the total number of individuals with dual comorbidity (*N* = 230))

	Hypertension/ hypercholesterolemia	Osteomuscular	Heart	Respiratory	Mental health	Diabetes/ thyroid	Neurological	Urinary	Gastrointestinal	Other
Hypertension/ hypercholesterolemia	—	23.9 (55)	10.4 (24)	6.1 (14)	3.5 (8)	3.9 (9)	3.5 (8)	2.2 (5)	1.7 (4)	3.9 (9)
Osteomuscular		—	5.2 (12)	5.2 (12)	7.4 (17)	2.6 (6)	0.9 (2)	0.9 (2)	1.3 (3)	2.6 (6)
Heart			—	2.2 (5)	2.2 (5)	1.3 (3)	1.3 (3)	0.0 (0)	0.4 (1)	0.0 (0)
Respiratory				—	0.0 (0)	0.4 (1)	0.0 (0)	0.9 (2)	0.0 (0)	0.4 (1)
Mental Health					—	0.4 (1)	0.0 (0)	0.4 (1)	1.7 (4)	1.3 (3)

**Appendix 2:** Secondary analysis examining the association between morbidity and prompt help-seeking (within 3 months from symptom onset) versus non-prompt help-seeking—Univariable analysis

Crude OR of prompt help-seeking (95% CI) *P*-value							
	Persistent cough or hoarseness (*n* = 235)	Persistent unexplained pain (*n* = 184)	Abdominal Bloating (*n* = 172)	Change in bowel habits (*n* = 142)	Change in bladder habits (*n* = 134)	Change in a mole or a new mole (*n* = 127)	Rectal bleeding (*n* = 117)
Comorbidity							
Osteomuscular	1.4 (0.8, 2.4) *P* = 0.19	(a)	1.8 (0.9, 3.6) *P* = 0.10	1.1 (0.5, 2.1) *P* = 0.87	1.0 (0.5, 2.0) *P* = 0.98	0.8 (0.4, 1.8) *P* = 0.65	1.2 (0.5, 2.6) *P* = 0.73
Hypertension/ hypercholesterolemia	**2.3 (1.3, 3.9) *P* = 0.003**	1.8 (1.0, 3.2) *P* = 0.06	**2.4 (1.2, 4.8) *P* = 0.02**	0.7 (0.4, 1.4) *P* = 0.34	1.1 (0.5, 2.2) *P* = 0.83	**2.3 (1.1, 4.7) *P* = 0.03**	1.6 (0.7,3.6) *P* = 0.25
Mental health	1.1 (0.5, 2.6) *P* = 0.79	(a)	(a)	0.5 (0.2, 1.1) *P* = 0.09	1.0 (0.4, 2.6) *P* = 1.00	0.9 (0.3, 2.9) *P* = 0.85	0.4 (0.1, 2.0) *P* = 0.28
Respiratory	(a)	0.6 (0.2,1.4) *P* = 0.22	1.1 (0.4, 2.9) *P* = 0.89	0.7 (0.2, 2.1) *P* = 0.51	0.7 (0.2, 2.4) *P* = 0.57	0.5 (0.1,1.6) *P* = 0.24	1.1 (0.3, 4.1) *P* = 0.84
Heart problems	0.8 (0.4, 1.6) *P* = 0.61	1.2 (0.6, 2.6) *P* = 0.58	1.0 (0.4, 2.3) *P* = 1.00	0.7 (0.3, 1.4) *P* = 0.30	1.0 (0.5, 2.3) *P* = 0.91	0.5 (0.2, 1.5) *P* = 0.23	1.8 (0.7, 4.4) *P* = 0.20
Diabetes/ Thyroid	1.2 (0.5, 2.9) *P* = 0.66	1.2 (0.5, 3.2) *P* = 0.67	(a)	1.3 (0.5, 3.5) *P* = 0.61	3.6 (0.8, 14.9) *P* = 0.08	1.0 (0.2, 6.0) *P* = 0.97	3.9 (1.0, 14.6) *P* = 0.05
Urinary	0.8 (0.3, 2.0) *P* = 0.57	1.3 (0.4, 4.9) *P* = 0.66	1.8 (0.5, 6.4) *P* = 0.37	1.0 (0.3, 2.8) *P* = 0.96	(a)	0.7 (0.1, 4.0) *P* = 0.70	**5.5 (1.5, 19.7) *P* = 0.01**
Neurological	2.0 (0.8, 5.1) *P* = 0.14	(a)	(a)	(a)	(a)	0.5 (0.1, 2.0) *P* = 0.34	0.9 (0.17, 4.84) *P* = 0.90
Gastrointestinal	0.4 (0.1, 1.8) *P* = 0.22	0.8 (0.3, 2.2) *P* = 0.59	1.5 (0.6, 3.9) *P* = 0.43	(a)	1.2 (0.3, 5.8) *P* = 0.79	3.0 (0.5,17.3) *P* = 0.21	(a)

Bold represents associations at *P* < 0.05.

(a) Excluded pairs where symptoms might relate to morbidity (e.g. ‘COPD-cough; osteomuscular-pain’)*.*
